# Wnt/β-Catenin Pathway in Experimental Model of Fibromyalgia: Role of Hidrox^®^

**DOI:** 10.3390/biomedicines9111683

**Published:** 2021-11-13

**Authors:** Ramona D’Amico, Marika Cordaro, Rosalba Siracusa, Daniela Impellizzeri, Angela Trovato Salinaro, Maria Scuto, Maria Laura Ontario, Roberto Crea, Salvatore Cuzzocrea, Rosanna Di Paola, Roberta Fusco, Vittorio Calabrese

**Affiliations:** 1 Department of Chemical, Biological, Pharmaceutical and Environmental Sciences, University of Messina, 98166 Messina, Italy; rdamico@unime.it (R.D.); rsiracusa@unime.it (R.S.); dimpellizzeri@unime.it (D.I.); rfusco@unime.it (R.F.); 2 Department of Biomedical, Dental and Morphological and Functional Imaging, University of Messina, 98125 Messina, Italy; cordarom@unime.it; 3 Department of Biomedical and Biotechnological Sciences, University of Catania, 95124 Catania, Italy; trovato@unict.it (A.T.S.); mary-amir@hotmail.it (M.S.); marialaura.ontario@ontariosrl.it (M.L.O.); calabres@unict.it (V.C.); 4 Oliphenol LLC., 26225 Eden Landing Road, Unit C, Hayward, CA 94545, USA; robertocrea48@gmail.com

**Keywords:** fibromyalgia, pain, WNT/β-catenin pathway, Nrf2 pathway

## Abstract

Fibromyalgia (FM) is a chronic condition characterized by persistent widespread pain that negatively affects the quality of life of patients. The WNT/β-catenin signaling pathway seems to be involved in central sensitization and different pain states. The objective of this study was to investigate the beneficial effects of a new compound called Hidrox^®^ (HD), containing 40–50% hydroxytyrosol, in counteracting the pain associated with FM. An FM-like model was induced in rats by subcutaneous injections of reserpine (1 mg/kg) for three consecutive days. Later, HD (10 mg/kg) was administered orally to the animals for seven days. Reserpine injections induced WNT/β-catenin pathway activation, release of pro-inflammatory mediators as well as a significant increase in oxidative stress. Daily treatment with HD was able to modulate the WNT/β-catenin and Nrf2 pathways and consequently attenuate the behavioral deficits and microglia activation induced by reserpine injection. These results indicate that nutritional consumption of HD can be considered as a new therapeutic approach for human FM.

## 1. Introduction

Fibromyalgia (FM) is a disorder characterized by widespread pain throughout the body, accompanied by other symptoms such as fatigue, sleep and mood disturbances, and cognitive dysfunction [1,2,3]. The heterogeneity of FM makes it difficult to understand its pathophysiology. Despite decades of research, the specific cellular and molecular mechanisms underlying chronic pain remain elusive, and clinical approaches for treating FM are limited. It is known that numerous processes are intricated in the onset of pain, including, among others, changes in sensory perception of pain, inflammation, and oxidative stress [4,5].

Wnt family proteins are known to play a critical role in several forms of chronic and neuropathic pain [6,7,8]. Currently, three Wnt signaling pathways have been identified; the canonical Wnt/β-catenin pathway is the best studied and appears to be involved in the pathophysiology of various CNS disorders [9,10,11]. In this pathway, Wnt ligands bind to the cysteine-rich domain frizzled (FZ) receptors which in turn activate several intracellular signaling cascades. At the intracellular level, Wnt/FZ require the intracellular β-catenin protein, whose levels remain low by the action of a so-called destruction complex [12]. Wnt signaling activation leads to the dissociation of the β-catenin destruction complex; as a consequence, β-catenin accumulates in the cytoplasm and translocates to the nucleus, finally inducing the expression of Wnt target genes [13,14]. Moreover, it was demonstrated that spinal blockade of WNT signaling prevents the onset and persistence of hyperalgesia and allodynia following neuropathic pain [15]. Furthermore, in the spinal cord, WNT signaling may contribute to microglial activation, the stimulation of BDNF release, and enduring changes in the properties of dorsal horn neurons that initiate central sensitization [9,16]. Therefore, we imagined that targeting the WNT/β-catenin pathway with natural molecules without side effects that would interfere with the analgesic drugs may be an efficient strategy to counteract FM-associated pain. Many studies have shown the positive effects of natural food phytocomponents and a Mediterranean diet (MD) in oxidative and painful diseases [17,18]. The Mediterranean diet promotes a high intake of fruits and vegetables, leading to the reduction of saturated fats. In particular, one of the main components of the Mediterranean diet is olive oil, and the main phytochemical contained in it is hydroxytyrosol (HT) [19]. Hydrox^®^ (HD), which contains 40–50% HT, has been described as a free-radical scavenger and an antioxidant with important antimicrobial, anti-inflammatory, and neuroprotective properties [20,21,22,23,24]. Due to the biological properties of HT and its safety profile [25], the focus of our study was to investigate whether HD was able to modulate nociception, microglia activation, and oxidative stress, using an FM-like model induced by reserpine injections.

## 2. Materials and Methods

### 2.1. Animals

Male Sprague Dawley rats (200–250 g, 5–7-week-old; Envigo, Milan, Italy) were used in this research. Rats were housed in individual cages (five per cage) and maintained under a 12:12 h light/dark cycle at 21 ± 1 °C and 50 ± 5% humidity. A standard laboratory diet and tap water were available ad libitum. This study was approved by the University of Messina Review Board for the care of animals. Animal care conformed to Italian regulations on the use of animals for experimental and scientific purposes (D.Lgs 2014/26 and EU Directive 2010/63).

### 2.2. Induction of Fibromyalgia

A fibromyalgia-like model was induced by reserpine injection (1 mg/kg, subcutaneous) for three consecutive days [26]. Reserpine (Sigma-Aldrich, St. Louis, MO, USA) was dissolved in distilled water with 0.5% acetic acid. Sham animals received the same volume of distilled water with 0.5% acetic acid, but no added reserpine.

### 2.3. Experimental Groups

The rats were randomly divided into the following groups (*n* = 15 for each):

- Vehicle: an FM-like model was induced in rats as described in the previous paragraph and they were treated with saline (orally, o.s.) for 7 days, starting from the day after the last reserpine injection.
- Vehicle + HD: an FM-like model was induced in the rats as previously described and they were treated with HD (10 mg/kg, o.s.) for 7 days, starting after the last reserpine injection.
- Sham: The rats received no reserpine administrations as described in previous paragraph and were treated orally with saline or HD for 7 days, starting from the day after the last vehicle injection. Since no significant changes were found between the groups, we present data from the sham + saline group.

The dose and route of HD treatment were chosen based on previous studies [26,27,28]. At the end of the experiment, the L4–L6 region of the spinal cord from each rat was collected for all analyses.

### 2.4. Western Blot Analysis

Western blot analysis was performed as previously described [19]. Cytosolic and nuclear extracts were divided. Brain tissues from each rat were suspended in an extraction buffer containing 0.15 μM pepstatin A, 0.2 mM phenylmethylsulfonyl fluoride (PMSF), 1 mM sodium orthovanadate, and 20 μM leupeptin, which was homogenized at the highest setting for 2 min and centrifuged at 1000× *g* for 10 min at 4 °C. Supernatants contained the cytosolic fractions, while the pellets contained the nuclear ones. Pellets were re-suspended in a second buffer containing 150 mM sodium chloride (NaCl), 1% Triton X-100, 1 mM ethylene glycol tetraacetic acid (EGTA), 10 mM tris–chloridric acid (HCl) pH 7.4, 0.2 mM PMSF, 1 mM Ethylenediaminetetraacetic acid (EDTA), 0.2 mM sodium orthovanadate, and 20 μm leupeptin. After centrifugation at 4 °C and 15,000× *g* for 30 min, the nuclear protein containing the supernatants were stored at −80 °C for further analysis [29]. The following primary antibodies were used: anti-Wnt3a (Santa Cruz Biotechnology (SCB), Dallas, TX, USA, sc-80457), anti-FZ8 (Bioworld Technology, St Louis Park, MN, USA), anti–β-catenin (BD Biosciences, Franklin Lakes, NJ, USA), anti-active β-catenin (Millipore, Burlington, MA, USA), anti-Nuclear factor erythroid 2-related factor 2 (Nrf2; SCB, sc-365949), anti-Heme Oxigenase 1 (HO-1; SCB, sc-136960), anti-NAD(P)H quinone oxidoreductase-1 (NQO-1; Cell Signaling Technology, Danvers, MA, USA), and β-actin (SCB, sc8432) and anti-lamin A/C (Sigma-Aldrich). Protein expression was quantified by densitometry with BIORAD ChemiDocTM XRS+software (Segrate (MI)—Italy) and normalized to β-actin or lamin A/C levels. Images of blot signals were imported to analysis software (Image Quant TL, v2003; Sigma-Aldrich, Hamburg, Germany) [30].

### 2.5. Immunohistochemical Analysis

Immunohistochemical analysis was performed as previously described [31]. Sections of spinal cord were incubated with the following primary antibodies: anti-ionized calcium binding adaptor molecule 1 (Iba1; Thermo Fisher Scientific, Waltham, MA, USA) and anti-Brain-Derived Neurotrophic Factor (BDNF; SCB, sc33673) antibodies. Images were collected using a Leica DM6 microscope (Leica Microsystems SpA, Milan, Italy) following a typical procedure. The histogram profile is related to the positive pixel intensity value obtained [32].

### 2.6. Enzyme-Linked Immunosorbent Assay (ELISA)

The supernatant of the homogenate of spinal cord tissue was centrifuged and operated [26,33]. The expression of interleukin (IL)-18, tumor necrosis factor (TNF)-α, and IL-1β were measured using ELISA kits (R&D Systems, Minneapolis, MN, USA) following the manufacturer’s instructions.

### 2.7. Behavioural Testing

In a separate set of experiments, 5 additional animals for each group were used for behavioral testing. The rats were placed in behavior rooms for 5 min for 2 days for acclimation prior to the start of behavioral testing. All behavioral tests were performed on day 0 and 3, 5, 7, and 10 post-first injection and were conducted by expert observers blinded to the study. Tests are described below:

#### 2.7.1. Von Frey Hair Test

The Electronic von Frey test (BIO-EVF4, Bioseb, Vitrolles, France) was used to evaluate mechanical allodynia, as previously explained [26]. All the results are expressed as the force, in grams, at which the rat removed its paw, indicated as the withdrawal threshold.

#### 2.7.2. Hot Plate Test

The hot-plate latency was assessed as previously described [5]. All the results are expressed as paw-withdrawal latency(s) and the maximal latency accepted was 45 s [34].

#### 2.7.3. Tail-Flick Warm Water Test

This test was used to evaluate spinal thermal sensitivity. The duration of the tail withdrawal reflex was recorded as previously described [26]. The cut off time was 10 s to minimize tissue damage to the tail.

### 2.8. Statistical Evaluation

All values are expressed as mean ± standard error of mean (SEM) of N observations. All images are representative of the last 3 experiments performed on diverse experimental days on tissue sections collected from each animal. For in vivo studies, *n* represents the number of animals used. The results were analyzed by one-way ANOVA followed by a Bonferroni post-hoc test for multiple comparisons. A *p* value less than 0.05 was considered significant.

## 3. Results

### 3.1. Effect of HD on WNT/FZ/β-Catenin Signaling Pathway

**Figure 1:**
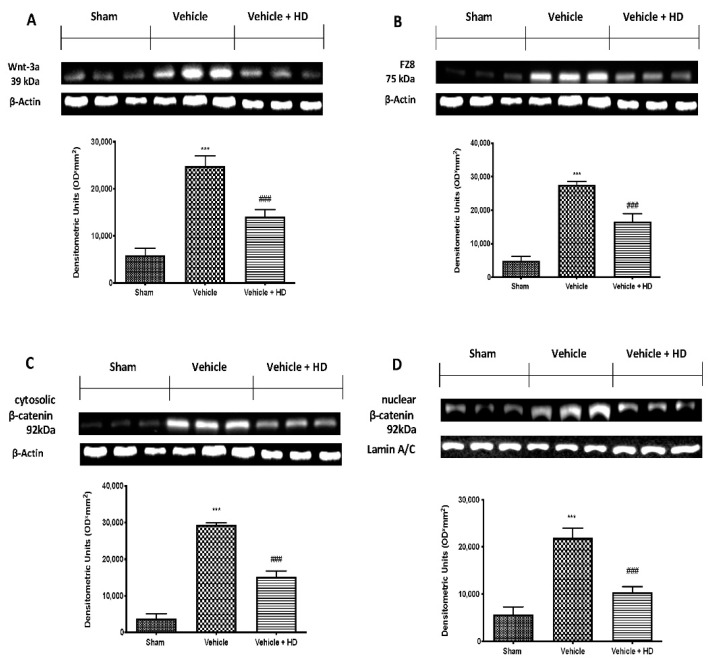
Effect of HD on WNT/FZ/β-catenin signaling pathway: Western blot analysis of Wnt-3a (**A**), FZ8 (**B**), cytosolic β-catenin (**C**) and nuclear β-catenin (**D**) expression. *** *p* < 0.001 vs. sham; ### *p* < 0.001 vs. vehicle.

We first addressed whether Wnt/β-catenin signaling in the spinal cord was altered following reserpine injections. Western blot analysis showed increased WNT3a (Figure 1A) and FZ8 (Figure 1B) in samples harvested from the vehicle group, compared to sham animals. HD administration significantly decreased both WNT3a and FZ8 expression (Figure 1A,B, respectively). Additionally, we determined the expression of β-catenin, a down-stream effector of the Wnt/β-catenin pathway. Our analysis showed an important increase in both cytosolic (Figure 1C) and nuclear (Figure 1D) fractions β-catenin expression. HD at a dose of 10 mg/kg strongly reduced β-catenin expression in the cytoplasm and nucleus.

### 3.2. Effect of HD on Pro-Inflammatory Cytokines Levels

**Figure 2:**
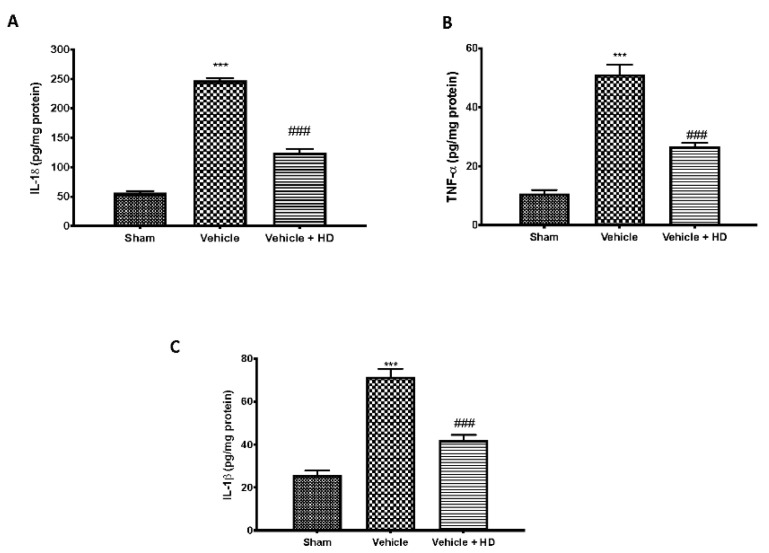
Effect of HD on pro-inflammatory cytokines levels: ELISA assay of IL-18 (**A**) TNF-α (**B**) and IL-1β (**C**) *** *p* < 0.001 vs. sham; ### *p* < 0.001 vs. vehicle.

WNT/β-catenin activation regulates the activity of the proinflammatory cytokines. Increased levels of IL-18 (Figure 2A), TNF-α (Figure 2B) and IL-1β (Figure 2C) were detected in the vehicle group, compared to sham animals. HD at a dose of 10 mg/kg significantly reduced levels of all cytokines.

### 3.3. Effect of HD on Mechanical and Thermal Hyperalgesia

**Figure 3:**
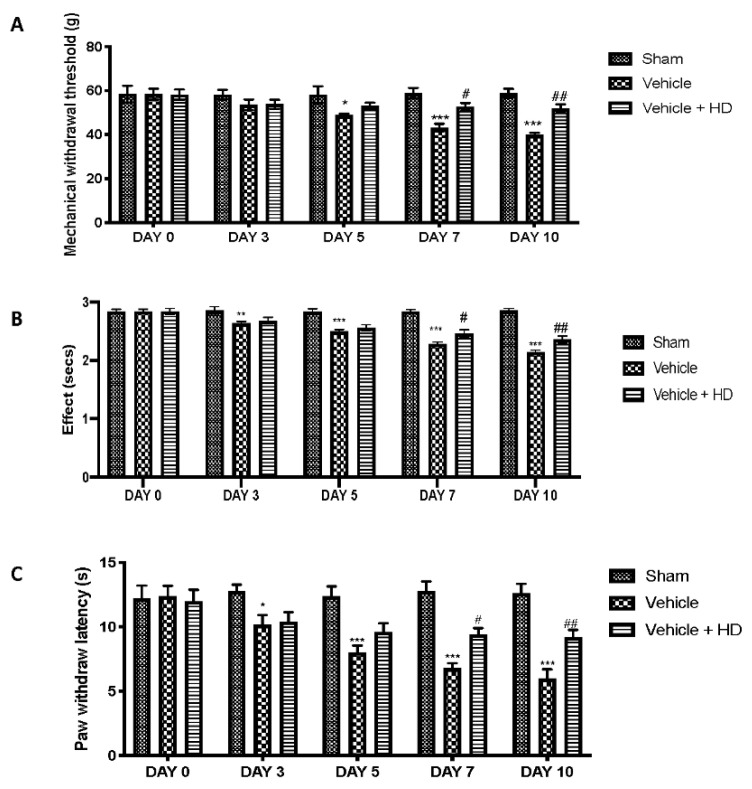
Effect of HD on mechanical and thermal hyperalgesia: von Frey hair (**A**) hot plate (**B**) and tail-flick warm water (**C**) tests. * *p* < 0.05 vs. sham; ** *p* < 0.01 vs. sham; *** *p* < 0.001 vs. sham; # *p* < 0.05 vs. vehicle; ## *p* < 0.01 vs. vehicle.

Behavioral tests were performed to investigate HD effect on mechanical and thermal hyperalgesia. Rats showed a reduction in the paw-withdrawal threshold in response to the von Frey test following subcutaneous injections of reserpine (Figure 3A). Additionally, vehicle rats showed increased pain sensitivity in the hot plate (Figure 3B) and tail-flick warm water (Figure 3C) tests. Oral administration of HD significantly reduced both mechanical allodynia and thermal hyperalgesia compared to the vehicle group.

### 3.4. Effect of HD on Microglia Activation and BDNF Expression

**Figure 4:**
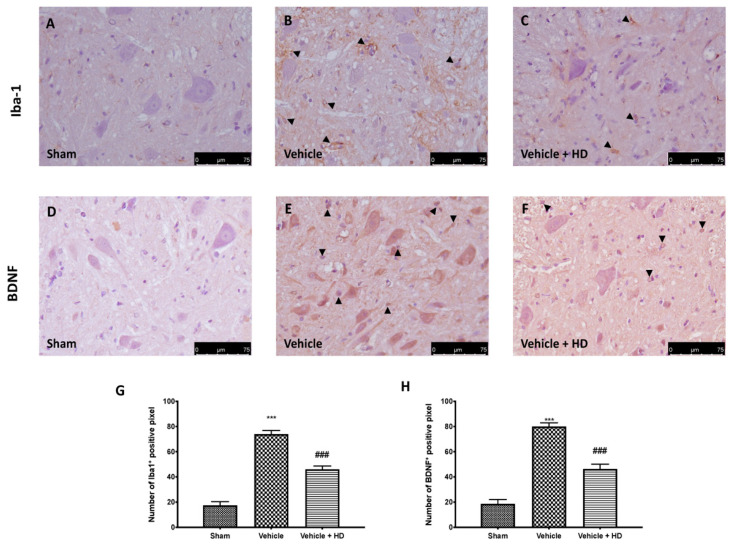
Effect of HD on microglia activation and BDNF expression: immunohistochemical evaluation in the spinal cord for Iba1 expression: Sham (**A**), Vehicle (**B**), Vehicle + HD (**C**) and BDNF expression: Sham (**D**), Vehicle (**E**), Vehicle + HD (**F**). Graphical quantification of Iba1 (**G**) and BDNF expression (**H**). The black arrows indicated positive cells. A 40× magnification is shown (75-µm scale bar). *** *p* < 0.001 vs. sham; ### *p* < 0.001 vs. vehicle.

The WNT/FZ8/β-catenin pathway is involved in microglia cell activation and release of BDNF from the microglia. Immunohistochemical analysis detected a significant increase in Iba1 expression in spinal cord sections from FM animals (Figure 4B), compared to sham rats (Figure 4A). In the same way, we detected an upregulation of BDNF expression following reserpine injections (Figure 4E), compared to controls (Figure 4D). HD treatment strongly reduces the number of positive cells for both Iba1 (Figure 4C) and BDNF (Figure 4F) compared to the vehicle group.

### 3.5. Effect of HD on Oxidative Stress

**Figure 5:**
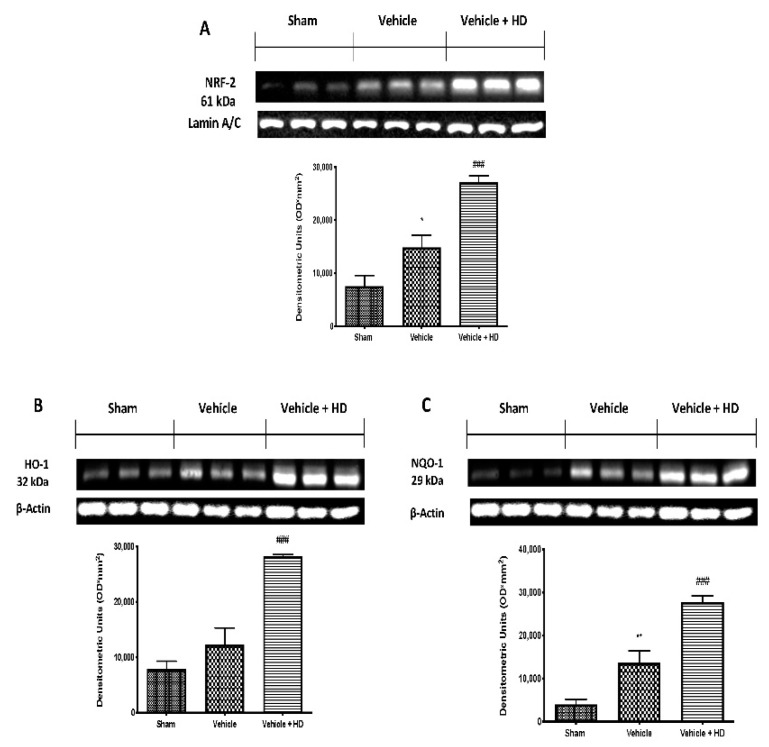
Effect of HD on oxidative stress: Western blot analysis of Nrf2 (**A**), HO-1 (**B**), NQQO-1 (**C**) expression. * *p* < 0.05 vs. sham; ** *p* < 0.01 vs. sham; ### *p* < 0.001 vs. vehicle.

It has been proven that oxidative stress is linked to the pathogenesis of FM [35]. Therefore, we investigated the Nrf2 pathway by Western blot analysis. Our results showed a small increase in Nrf-2 expression in the vehicle group compared to the sham group. A considerable increase in Nrf2 expression was induced by HD administration (Figure 5A). Consequently, Western blot analysis showed basal HO-1 (Figure 5B) and NQO-1 (Figure 5C) expression in the sham group. By contrast, the vehicle-treated group showed little increased HO-1 and NQO-1 expression, which were upregulated in HD-administered animals. 

## 4. Discussion

FM is a complex, painful disorder that necessitates a multidisciplinary approach for its therapy [1]. The current drugs for treating FM symptoms include many classes of sedatives, analgesics, and antidepressants [36,37,38]. Nevertheless, not all are well tolerated and they do not cover the full range of symptoms associated with FM [39], thus making it necessary to develop new treatments. Several studies have shown that FM induces many molecular mechanisms that involve inflammation and oxidative stress [5,40]. Therefore, the aim of our study is to investigate the molecular mechanism induced by HD administration in an FM-like model. In particular, we explored the WNT/β-catenin signaling pathway activation in the spinal cord.

Wnt signaling was originally believed to be only involved in the regulation of cellular processes such as differentiation and migration during neuronal development [41]; nevertheless, recent research has shown that the Wnt pathway is also involved in the pathogenesis of neuropathic and bone cancer-induced pain [12,42]. Previously, it has been demonstrated that HT may exert an inhibitory role on Wnt/β-catenin signaling on breast cancer stem cells and the migration capacity of triple-negative breast cancer (TNBC) cell lines [43]. In light of the above, we wanted to investigate whether HD, with its modest amount of HT, was able to modulate WNT/β-catenin activation in an FM-like model in rats. Binding of WNTs to their FZ receptors activates the canonical WNT/β-catenin pathway [8], that, in turn, can activate intracellular signaling pathways [44]. We focused on active β-catenin, a key downstream effector of the Wnt/β-catenin pathway and evaluated its expression in the spinal cord. Western blotting showed substantial upregulation of both cytoplasmic and nuclear β-catenin expression at the level of L4–L6 in the dorsal spinal cord after three days of reserpine injections. In the same way, our molecular analysis showed increased WNT3a and FZ8 expression in the lumbar spinal cord. HD treatment reduced WNT3a and FZ8 expression and β-catenin accumulation in the cytosolic and nuclear compartments. While upstream activation of β-catenin is triggered by WNT-binding FZ receptors, downstream WNT/β-catenin activation regulates the activity of proinflammatory mediators, such as IL-18, TNF-α and IL-1β, which may directly contribute to the onset and persistence of pain [45,46], as demonstrated by behavioral alterations. HD showed important anti-inflammatory activities by reducing IL-18, TNF-α and IL-1β levels, downregulating the inflammation associated with pain. These results were supported by behavioral tests. After HD administration, rats showed reduced pain sensitivity in mechanical allodynia and thermal hyperalgesia. Our data are in line with the literature, which reports that activation of WNT/β-catenin signaling contributes to increasing pain sensitivity [46]. Next, we explored the mechanism underlying pain development triggered by the activation of Wnt signaling. Immunohistochemical analysis showed a reduction in the number of positive cells for Iba-1 after HD treatment, whose expression was upregulated after reserpine injections. Furthermore, BDNF released by spinal microglia is involved in the pathogenesis of chronic and neuropathic pain [47]. Previous studies have proven that BDNF levels in the spinal cord are increased in different nerve injury-induced neuropathic pain models [9]. Consistent with this, we confirmed that BDNF expression significantly increased in spinal cords with FM, while it decreased in a significant manner after oral administration of HD. These results suggest that spinal modulation of the WNT/FZ8/β-catenin signaling pathway prevents activation of microglial cells and BDNF release from microglia.

Additionally, ROS overproduction by microglia is suggested to be a main cause of neuronal damage and dysfunction inducing derangement of neuronal redox signaling circuits or direct oxidative damage [27,48]. Much evidence supports the role of oxidative stress in the progress of FM [5,49]. Hidrox^®^ is known to influence the promotion of the transcription of genes downstream of Nrf2 activation [22]. Nrf2 represents a key regulator of cellular antioxidant response [50,51]. Under the physiological circumstances, Nrf2 is inhibited in the cytoplasm by binding with Keap1. However, Nrf2 separates from Keap1 and translocates to the nucleus to bind to ARE upon stimulus or oxidative stress. Activation of the Nrf2-ARE pathway has a protective effect against various diseases via antioxidative mechanisms [50]. Nrf2 signaling is important for maintaining antioxidant/oxidant homeostasis and for defending against ROS by modulating a variety of protective enzymes, including HO-1 and NQO-1, all of which have strong antioxidant properties [50]. Here, we displayed that HD administration upregulated the Nrf2 transcriptional system, inducing the activation of phase II detoxifying enzymes, such as HO-1 and NQO-1, thus contributing to reduced pain-like symptoms.

## 5. Conclusions

In conclusion, these results demonstrate that WNT signaling was broadly activated in the nociceptive pathways in the spinal cord after reserpine-induced FM. Here, we demonstrate that HD was able to modulate activation of the WNT/-catenin signaling pathway. Furthermore, we confirm the anti-inflammatory and antioxidant properties of HD, as shown by a reduction in pro-inflammatory cytokines, microglia activation, and oxidative damage.

## Data Availability

The data used to support the findings of this study are available from the corresponding author upon request.
